# Supplementary material for: Are carbon emissions trading and green financial instruments synergistic? -Comprehensive quantitative research based on content analysis
Source: PLoS One. 2024 Mar 7;19(3):e0298601. doi: 10.1371/journal.pone.0298601 (PMC10919601; doi:10.1371/journal.pone.0298601)
Supplement: S1 Data — (ZIP) [file pone.0298601.s001.zip › Data/地级市Z_F工作报告.zip!/░▓╗╒/┬φ░░╔╜╩╨/└┤╫╘╣1⁄2╓┌║┼í╛┬φ┐╦╩2╛▌═°í┐_2012.docx]

# **政府工作报告（2012）**

【字体大小：[大](http://zwgk.mas.gov.cn/openness/detail/content/javascript:doZoom(18)) [中](http://zwgk.mas.gov.cn/openness/detail/content/javascript:doZoom(15)) [小](http://zwgk.mas.gov.cn/openness/detail/content/javascript:doZoom(14))】

政府工作报告

——2012年1月6日在马鞍山市第十四届

人民代表大会第六次会议上

市长张晓麟

各位代表：

　　现在，我代表市人民政府，向大会作工作报告，请予审议，并请市政协委员和其他列席人员提出意见。

一、2011年政府工作回顾

　　刚刚过去的2011年，是实施“十二五”规划的第一年，是我市行政区划大调整之年，也是应对挑战攻坚克难、改革发展取得显著成效的一年。在省委、省政府和市委的正确领导下，市政府团结带领全市人民，以科学发展观为统领，以加快转变经济发展方式为主线，以推动皖江示范区建设为抓手，全力推进大规划、大建设、大发展，较好地完成了市十四届人大五次会议确定的各项目标任务，实现了“十二五”发展的良好开局。

　　预计全市实现地区生产总值1090亿元，比上年增长12.3%；财政收入186.3亿元，增长22%；全社会固定资产投资1230亿元，增长38%；社会消费品零售总额225.7亿元，增长17.5%；城市居民人均可支配收入27330元，增长18%；农民人均纯收入9220元，增长15%。

(一)经济发展呈现多点支撑格局

　　工业、农业、服务业平稳较快增长。深入开展“服务企业促进发展”活动，全市规模以上工业实现增加值550亿元，增长14.5%。新增规模以上工业企业123户，总数1036户(按新口径，新增53户，总数573户)；新增销售收入50亿元以上企业1户，10亿-50亿元企业3户。马钢重组长钢，组建设立矿业、环保、物流、财务公司，实现销售收入900亿元。建筑业总产值240亿元，增长30%。十七冶主营业务实现多元发展。农业生产取得好收成。新增农村土地流转面积7.5万亩，总面积49.3万亩。当涂农业示范区晋升为省级现代农业示范区，市开发区食品加工园区晋升为省级农业产业化示范区，全市农产品加工产值突破200亿元。服务业实现增加值300亿元，增长8.5%。主城区核心商圈聚集辐射功能增强。旅游业总收入67.8亿元，增长87%。我市成为省级服务外包示范城市，服务外包产值增长1.5倍。和县蔬菜博览会暨现代农业项目推介会、当涂民歌艺术节暨石臼湖螃蟹节、博望刃模具暨机床博览会等一批品牌会展成为服务业发展亮点。全市新发展私营企业3212户，总数1.55万户；新发展个体工商户1.09万户，总数6.83万户。

　　县区、园区、新区发展齐头并进。三县生产总值平均增长16%，高于全市增幅3.7个百分点；财政收入平均增长50.5%，高于全市增幅28.5个百分点。三区主要经济指标跻身全省城区前10位；财政收入两年翻一番，增幅均在47%以上。市开发区一重高端装备制造、开米绿色清洗剂等项目开工建设，圆融光电等项目竣工投产，郑蒲港新区组建成立；博望新区规划建设全面展开，筹建省级高新区获省政府批准；慈湖高新区启耀光电、中海新材料等项目竣工投产，国家级高新区申报工作通过科技部现场考核评审；示范园区新注册公司218家，中弘光伏等15家规模以上企业建成投产；滨江新区沿江大道、采石河路全线贯通，长江湿地文化公园等项目签约；秀山新区东部环路主体工程完工，6家知名软件企业落户软件园。六个园区、新区累计完成投资434亿元，占全市固定资产投资的35.3%。

(二)转型发展迈出新步伐

　　产业层次加快升级。实施工业企业升级行动计划，全市322个重点技改项目完成投资235.7亿元，增长41.6%。马钢新区二期结构调整工程全面展开，高速车轮技改项目投入试生产。华菱3万辆重卡底盘项目竣工投产。铁基新材料高技术产业集群发展规划获省政府批准，高端装备制造、电子信息、光伏光电、节能环保等新兴产业快速发展，全市战略性新兴产业完成投资249亿元，增长45%；实现产值230亿元，增长40%。新增高新技术企业39家、高新技术产品110个，高新技术产业增加值占规模以上工业增加值31.6%。马钢被列为国家创新型企业，“梦都”、“金菜地”被授予“中国驰名商标”，“采石矶”茶干被评为“中华老字号”。3项发明专利获中国专利优秀奖。科达洁能院士工作站组建。我市再次荣获“全国科技进步先进市”称号，国家知识产权试点市创建工作通过考评验收。集约发展加速推进。建立土地集体决策制度，启动土地“四模创建”活动，开展国土“三项行动”，顺畅有序的土地管理机制初步形成。当年获批用地3.19万亩，是上年的3倍；新增补充耕地1.03万亩；含山县成为“全省土地整治示范县”。征迁拆迁拆违强力推进，拆迁面积390万平方米，交付土地3.7万亩，拆除城乡违法建设39万平方米。

　　节能减排扎实有效。马钢煤气综合利用发电工程等重点节能项目竣工投入运行。一批落后产能被淘汰。电力迎峰度夏工作综合考评居全省首位。公共机构节能活动深入开展。实施36项减排重点工程，5座污水处理厂外部管网配套一期工程基本竣工。全市单位生产总值综合能耗下降5.5%，主要污染物排放量控制在省下达的指标以内，在全国城市环境管理和综合整治考核中名列全省第一。

(三)体制机制改革深入推进

　　重点领域改革取得突破。深化完善投融资管理体制改革，初步建立“借、用、管、还”一体化，决策、管理、执行、监督相分离的投融资管理体系；重组成立城市发展投资集团，创新发展模式和融资方式，融资平台进一步夯实，融资渠道明显拓宽，融资规模持续扩大。深化完善工程建设管理体制改革，实施工程建设项目“六分开”，对每个工程实行“四合一”计划管理；组建重点工程建设管理局；对政府投资项目实行“两统一表”制度；整合市本级土地、房屋征迁管理机构，成立市土地和房屋征收管理局；进一步探索完善招标采购管理体制。深化完善国有资产管理体制改革，健全国资委议事制度，加强国有资产投资管理和业绩考核。对市直行政事业单位国有房地产实施产权统管，闲置房产统一实行市场化招租拍卖。深化完善财税管理体制改革，健全协税护税工作机制。

　　金融工作创新力度持续加大。制定银行业机构考核办法，与我省5家银行相继签署战略合作协议。招商银行、光大银行马鞍山分行开业运营，民生银行马鞍山分行获准筹建，银行业金融机构增至18家。马鞍山农商行跨区新设立村镇银行6家、支行2家。新设小额贷款公司18家、融资性担保公司9家、典当行2家、创业投资基金2支，4家证券营业部、2家期货营业部开业运营，网上金融超市开通。20亿元企业债券发行通过初审，中小企业集合票据发行工作积极推进。金融贷款余额791亿元，新增160亿元；银行承兑汇票120亿元；小额贷款公司投放22.5亿元；资本市场直接融资97.6亿元，社会融资总规模1150亿元，为地方经济社会发展提供了有力支持。

(四)开放层次水平明显提升

　　招商引资取得新成果。强化招商引资工作市级统筹，完善考核办法，建立信息共享机制，成立招商政策协调小组和专业招商小组。改进招商办法，推动产业招商、专业招商和无地招商。举办新区新项目、文化旅游、高端装备、产权要素专题推介会，组团参加中博会、徽商大会、绿展会、农交会等活动。落实皖粤经贸合作部署，与中山市签署全面加强经贸合作框架协议；与央企、全国知名民企对接合作取得明显成效。全市实际利用内资830亿元，增长69.7%；实际利用外资9.81亿美元，增长20.4%。

　　对外合作交流继续深化。精心组织“经贸文化宝岛行”活动，10个项目签约，总投资15.1亿美元；与花莲县、新竹市签订合作协议，在台设立全省首个经贸文化交流中心。成功承办第四届全球外包大会、“10 + 1”中国-东盟贸易谈判委员会会议、“10+3”东亚自由贸易区研讨会等国际性会议。全年实现进出口总值39亿美元，增长28.3%。外籍轮直航102艘次，口岸外贸运量增长12.2%。推进宁马一体化、马芜同城化取得新进展，宿马经济区建设进展顺利。

　　(五)城乡承载功能进一步增强

　　基础设施建设加快推进。全年实施城乡基础设施项目480个，完成投资310亿元。长江公路大桥建设进度加快，当年完成投资13.6亿元。宁安城际铁路主体及配套工程、芜申运河当涂段航道整治稳步推进。东环路原址改造基本完成，改线工程加紧实施。205国道马鞍山南段升级改造工程正式动工，314省道改建路基工程、105省道改建主体工程基本完成。马濮一级公路、沿江大道北段建成通车，湖西南路、花山路改造工程完工。人头矶港区主体工程竣工，太平府航道正式开通。市体育会展中心加快建设，“三馆”主体工程完工。小城镇建设步伐加快。姑孰凌云大桥、年陡龙山大道建成通车。升级改造县乡道路91.3公里、通村道路53.6公里，危桥改造项目全面实施。

　　环境面貌明显改观。慈湖河中游综合整治工程开工建设，花雨广场改造及南湖景观提升一期工程完成。实施18个老旧小区整治，完成沿街主干道门头招牌提档改造和两侧楼房立面出新，加大水系整治和街头游园建设力度。2700户农村危房改造完成，9个乡镇垃圾中转站完工，农村环境连片整治启动。江心洲段河道整治工程全面完成，23座水库除险加固主体工程基本竣工，石臼湖湖口水利枢纽工程前期工作开展。防汛抗旱扎实有效，全省农田水利基本建设现场会在和县召开。

　　(六)社会建设和管理稳步推进

　　民生工程深入实施。45项民生工程投入资金24.6亿元，增长29%。期末城镇登记失业率2.9%。被征地农民养老保险、新型农村养老保险、企业退休人员养老金、失业保险标准提高，城乡低保差距缩小。市社会福利中心一期工程建成使用。4.1万套安置房开工建设，2.3万套竣工交付，基本实现去年初提出的“力争让过渡期满18个月的征迁户在春节前搬入新居”目标。开工建设2.4万套各类保障性住房，完成省政府下达目标任务。稳定物价工作取得成效。认真贯彻落实国家调控政策，房地产市场总体平稳。扩大住房公积金缴存覆盖面，公积金归集和使用规范有序。公交优先发展战略继续实施。

　　社会事业全面进步。扎实开展“四城同创”活动，蝉联“全国文明城市”、“国家卫生城市”、“全国双拥模范城”荣誉称号，全国文明城市复查得分位居地级市第4名；“国家环保模范城市”复查通过省级预评。我市被中宣部等八部门评为“全国文化体制改革先进地区”，成为首批国家公共文化服务体系示范区创建城市；凌家滩被列入国家“十二五”大遗址保护规划，市文化馆、档案馆晋升国家一级馆；成功举办马鞍山中国李白诗歌节，“江南之花”、“周末大舞台”等群众性文化活动精彩纷呈。23所公办幼儿园改造建设工程全面实施，校安工程建设走在全省前列，当涂二中新疆班开班，马鞍山师专通过省人才培养模式评估，职业技术学院、技师学院职教资源实现整合，微软IT学院挂牌成立。雨山区基层医改经验在全省推广；市立医疗集团荣获全国公立医院改革创新奖，鼓楼医院集团秀山医院开工。成功举办市第十届运动会。人口和计划生育工作全面推进，低生育水平持续稳定。第六次全国人口普查圆满完成。金家庄区被授予“全国科普示范区”。外事、侨务、地方志工作继续加强，地震、气象、防灾减灾工作扎实有效。老年、青少年和妇女儿童工作取得新进展，老干部活动中心建成使用，花山区被授予“全国家庭教育先进区”。残疾人和红十字事业取得新进步。

　　积极探索社会管理。深入开展“精品”、“四优”社区创建活动，全面推行社区网格化管理。市社区便民服务网络中心挂牌运行，居家养老志愿服务在花山区率先开展。加强社会治安综合治理，刑事案件发案率下降4%，现行命案全部破获，各项严打专项整治行动战果显著。开展信访综合治理，畅通信访渠道，搭建三级平台，推行双向问责，信访秩序明显好转。双岗等7个小区供电用电秩序综合整治顺利完成，医患纠纷、道路交通事故赔偿第三方调处机制进一步完善。产品质量和食品药品安全监管力度加大。安全生产形势稳定。“六五”普法全面启动。国防动员、人民防空工作得到加强。民族团结、宗教和睦局面进一步巩固。

　　在推进经济社会发展的同时，我们不断加强政府自身建设。认真执行市人大及其常委会决议、决定，向市人大常委会报告工作和向市政协通报情况，主动接受市人大的法律监督、工作监督和市政协的民主监督。注意倾听和吸纳各民主党派、工商联、无党派和社会各界人士的意见建议。密切联系群众，“大走访”和“以人为本、执政为民”主题教育活动扎实开展。4项(9件)人大议案、98件建议批评意见、256件政协提案全部办复。建立市政府常务会议学法、解读政策制度，调整充实市政府法律顾问组，完善政府法律事务处理机制，制定政府重大行政决策程序规定，扎实推进行政复议示范工作。建立行政审批网上预审机制，政务公开不断深化。建立每月经济形势分析会制度、每周一市长碰头会例会制度，推行工作日志、工作反省点评制度。各项工作坚持高标准、严要求，省内争一流、全国争先进。强化目标考核，加大督办力度，有效保障了工作落实。实施审计监督“双百”制度。加强财政收支预算管理，全面清理财政专户和市直行政事业单位银行账户，推行公务卡结算制度。开展党政机关公务用车专项治理，实施公共权力规范运行预警机制，惩治和预防腐败体系建设深入推进。

　　各位代表！过去的一年，在宏观环境偏紧、市场急剧变化、转型压力加大的背景下，地区生产总值、固定资产投资双双超千亿，7项主要指标增幅超长三角城市平均水平，其中3项指标增幅位居前列，迈入了加速转型、加快发展的新阶段；我省部分行政区划调整，含山、和县划入马鞍山，我市进入了一江两岸、拥江发展的新时期；重点领域改革全力推进，服务大规划、大建设、大发展的体制机制更加顺畅；社会建设管理不断加强，民生持续改善；干部思想观念明显转变，解决问题的能力和水平不断提升。回顾总结过去的2011年，的确是马鞍山发展史上非同寻常的一年，是奋进年、发展年、改革年、民生年。

　　通过一年来的生动实践，我们深切体会到：必须坚持以科学的理念指导实践，紧抓发展第一要务不放松，以发展促转型，以转型谋发展，牢牢把握发展主动权；必须坚持解放思想、更新观念，发现新情况及时研究，发现新问题及时破解，发现新典型及时推广，增强工作的有效性和创造性；必须坚持用改革的办法解决前进中的问题，针对影响和制约发展的瓶颈因素，从一切有利于发展、有利于工作出发，构建充满活力的体制机制；必须坚持群众利益至上，把保障和改善民生作为政府一切工作的出发点和落脚点，真心亲民，真情为民，真干富民；必须坚持狠抓工作落实，重点查“不落实”的问题，集中抓“不落实”的环节，一抓到底，水落石出，做到件件有着落、事事有回音。

　　成绩来之不易，经验弥足珍贵。这是我们按照中央和省委、省政府的决策部署，在市委的坚强领导下，在市人大、市政协的监督支持下，在社会各界的大力帮助下，全市各级政府依靠广大人民群众，同心协力、砥砺奋进的结果。在此，我代表市人民政府，向辛勤工作在全市各条战线上的广大干部群众，向市人大代表、市政协委员，向各民主党派、工商联、人民团体和社会各界人士，向中央和省驻马单位、来马投资企业，向驻马解放军、武警官兵和公安民警，表示衷心的感谢并致以崇高的敬意！

　　在肯定成绩的同时，我们也清醒地认识到，我市经济社会发展中还存在不少困难和问题，政府工作与群众的新期待还有差距。主要是：受外部环境的影响，部分企业生产成本上升、经营困难、效益下滑，在手大项目、好项目不多，新兴产业尚处在培育期，节能减排形势不容乐观，经济发展、产业转型面临较大压力；面对区划调整新形势，优化城市布局、完善城市功能、加快城市转型发展的任务十分艰巨；社会转型期矛盾凸显，公共服务和社会保障水平有待提高，加强和创新社会管理还有大量工作要做；少数机关干部市场意识、忧患意识不强，行政效能不高、工作落实不力的现象还不同程度地存在。对此，我们要认真对待，积极采取措施加以解决。

二、2012年主要目标任务安排

　　今年是党的十八大召开之年，是我市全面贯彻落实市第八次党代会精神的第一年，也是本届政府任期的最后一年。做好今年政府工作，意义十分重大。中央明确了宏观经济政策“稳中求进”的工作总基调，更加突出分类指导、有扶有控的调控导向，这对我市转型发展、加快发展总体有利。市第八次党代会提出，要抓好加快转变经济发展方式、推动一江两岸协调发展、建设更高水平小康社会“三件大事”，建设重工业制造中心、江海联运枢纽中心、运动健康休闲中心和绿色食品供应中心“四个中心”，开启了马鞍山科学发展的新征程，为做好政府工作指明了方向。但也要看到，宏观形势总体仍然偏紧，各类风险明显增多，区域竞争更趋激烈，我市转型发展、加快发展的压力更加显现。我们既要增强危机意识，又要坚定发展信心，善于在变局中把握机遇，在逆境中主动作为，在挑战中勇于胜出。今年政府工作总体要求是：以邓小平理论和“三个代表”重要思想为指导，深入贯彻落实科学发展观，全面贯彻中央和省、市委的重要决策部署，紧紧扭住转变经济发展方式这条主线，坚持稳中求进、好中求快，更加突出工业兴市、产业转型、多点支撑，更加突出项目引领、开放带动、改革创新，更加突出两岸联动、城乡一体、民生改善，全力实施“项目推进年”活动，为抓好“三件大事”、建设“四个中心”打下坚实基础。

　　综合考虑，今年经济社会发展的主要预期目标为：地区生产总值增长12%；财政收入与地区生产总值同步增长；全社会固定资产投资增长25%；社会消费品零售总额增长16%；城市居民人均可支配收入、农民人均纯收入增长13%；城镇登记失业率控制在4%以内；人口自然增长率、单位生产总值能耗、主要污染物排放量控制在省下达指标以内。

　　为实现上述目标，我们将认真做好以下八个方面重点工作：

　　(一)全力实施“项目推进年”活动，促进经济平稳较快增长。项目是经济工作的总抓手。把今年作为“项目推进年”，抢抓国家扩大内需的政策机遇，进一步完善项目建设推进机制，继续推进省“861”行动计划项目，强力实施市重点项目500个以上，力争完成投资600亿元。

　　加强项目谋划。围绕国家投资政策导向，结合我市实际，在重大基础设施、战略性新兴产业、先进制造业、现代服务业等重点领域谋划储备一批牵动性强的大项目、好项目，及时充实完善重点项目库。对已上报的项目，加强联系衔接，努力在国家项目计划中争取更大份额，力求更多的项目落地开工。更加突出项目投资的先导性、效益性和基础性，把项目建设与优化产业结构、提升基础承载能力、改善民生结合起来，科学安排政府投资项目实施时序，增强项目建设对经济增长的拉动力和支撑力。全力保障项目落地。加强统筹协调，千方百计解决制约项目建设的土地、征迁、资金问题。按照“强有力保护耕地、高水平保障发展”的要求，深入开展国土“三项行动”和“四模创建”活动，继续做好建设用地报批和农村土地整治工作，切实保障项目用地需求。完善集体土地和房屋征收政策，进一步加大征迁拆迁拆违推进力度，直面困难，做细工作，确保拆得掉、拆得快、拆得稳。坚持争取上资、招商引资、激活民资、扩大融资“四资并举”，多渠道筹措项目建设资金，确保融得来、成本低，用得好、效益高。强化项目推进机制。分类排出竣工、续建、开工、前期工作项目，落实个人领衔负责制，加强项目协调调度。建立项目推进联动机制，着力优化项目审批、落地与开工程序，提升项目审批、用地报批、环境评价效能。实行项目全程跟踪、全速推进，促进项目快开工、快建设、快竣工。完善项目建设开竣工通报制度，强化重大项目考核，建立健全重大项目责任追究制，确保项目建设取得实效。

　　(二)加快产业转型升级，提升工业经济规模和层次。围绕打造“国内重要的重工业制造中心”，坚持工业兴市不动摇，抓增量扩存量，努力营造崇尚创业、尊重企业家的社会文化，加快形成“一马当先、万马奔腾”的产业发展新格局。力争新增规模以上工业企业超100家，规模以上工业增加值增长14%以上，突破600亿元。

　　推动优势主导产业做大做强。支持马钢做精钢铁主业，做强做大非钢产业，深化内部改革与管理，在全省率先成为销售收入超千亿元企业。推动华菱重卡进一步开拓国内外市场，积极开发核心零部件。开工建设山鹰80万吨纸等一批重大工业技改项目。深入实施工业企业升级行动计划，支持方圆支承、泰尔重工、三联泵业等一批中小企业发展壮大。全年新增上市公司3家。深入开展服务企业活动，有针对性地帮助企业解决资金、用工、用地等方面难题，积极做好地产品促销工作。

　　培育壮大战略性新兴产业。全力实施铁基新材料高技术产业集群发展规划，以高端装备制造、汽车、节能环保产业为重点，拉长产业链，扩大产业群。加快一重高端装备制造、亚重装备、科达洁能等45个重点装备制造业项目建设，推进金沙江光电产业园、晶能光电等19个重点光伏光电项目，建设数字硅谷国际产业园、中钢天源电子科技产业园等26个重点电子信息项目，加快形成新兴产业的市场规模优势和技术领先优势，力争战略性新兴产业产值增长40%以上。

　　强化科技创新支撑。突出企业创新主体地位，多渠道加大自主创新投入。加快合工大(马鞍山)高新技术研究院建设，深入推进院士工作站和产业技术创新战略联盟创建工作，积极与更多的高校和科研院所开展产学研对接合作。全年新认定高新技术企业25家、高新技术产品100项以上，实现高新技术产业产值530亿元。加大专利申报力度，确保专利授权量1200件以上。全力实施商标战略，至少培育中国驰名商标1个、省著名商标15个。促进资源节约、环境友好。完善工作机制，加强重点节能工程建设，推动现有高耗能行业技术进步和企业节能改造。加大减排工作力度，加快5座污水处理厂外部管网配套二期工程建设，当涂电厂脱硝工程、山鹰造纸污泥焚烧发电项目竣工运营。加强财税、金融等政策支持，积极发展循环经济。强化监督管理，进一步提升环境质量，确保通过国家环保模范城市复查验收。

　　(三)实施一江两岸联动，加快推进城市拥江发展。围绕建设“皖江地区中心城市”，进一步深化“1255”城市发展战略，启动城市总体规划修改工作，编制综合交通、长江港口及岸线利用等专项规划，以重大交通基础设施为先导，加快“一江两岸”协调发展。全年实施城乡建设项目600个以上，完成投资400亿元以上。

　　大力推进城市组团发展。以主城区为中心、副城区为两翼、中心镇为呼应，全面推进城市规划建设。进一步完善秀山新区路网框架，快速推进秀山湖、学校、医院等公共配套设施建设；加快滨江新区长江湿地文化公园建设，启动采石河环境综合整治、采石古镇改造项目；加快推进当涂姑孰新城建设、江心洲整体开发、和县历阳东进南拓，向主城区集聚。加快推进东接南京的博望副城区核心区建设，推进西接合肥的环峰副城区南拓西延，加强林头、运漕、香泉、石桥、黄池5个中心镇建设，不断增强副城区和中心镇承载发展、集聚人口、公共服务功能。三县要调整完善投融资、建设等体制机制，迅速掀起大规划、大建设热潮。

　　加强重大交通设施建设。围绕构建一江两岸快速交通网，加快长江公路大桥建设，同步推进出入口配套设施工程；着手谋划第二、第三过江通道。加快推进合马巢高速公路建设，启动建设马滁高速公路，开工206省道、226省道县段改建工程。进一步畅通博望副城区对接南京、连接主城区的快速通道，314省道升级改造竣工通车，推进313省道改建和博宁大道、宁丹大道前期工作。推进宁安城际铁路马鞍山段、京福高铁含山段、市客运中心枢纽站建设，抓紧与南京共同谋划城市轨道交通并完成项目报批。东环路高速化改造竣工通车。完成205国道马鞍山南段路基工程，开工建设北段示范工程。加快芜申运河当涂段航道整治，做好姑溪河、青山河航道提升工程前期工作。

　　着力提升城市品质。坚持系统规划、成片推进，注重单体建筑景观效果和整体协调性，加大旧城改造力度。打通城区“断头路”，实施破损道路综合改造提升，畅通城市微循环。启动雨山湖环湖景观改造规划，加快城市出入口、慈湖河综合整治步伐，建设雨山河防护林带，做好全国绿化模范城市复查迎检工作。加强中心城区公共停车场、集贸市场规划与建设，开展“三车”交通安全和营运综合整治，推进公厕标准化改造，规范户外店面广告，全面推行数字化城市管理。

　　(四)深化改革开放，不断增强发展活力。巩固扩大改革成果，推动重点领域改革向县区延伸覆盖，建立上下一体、顺畅有序的体制机制。发挥皖江示范区平台作用，进一步提升对外开放水平，全年实际利用外资突破10亿美元，增长10%以上；实际利用内资突破1000亿元，增长25%以上。

　　纵深推进各项改革。加快事业单位人事管理和分配制度改革步伐，积极稳妥推进事业单位分类改革。继续深化行政管理体制改革。抓住国家开展税制改革试点、地方债发行的有利时机，做好基础准备工作。深化预算编制与管理改革，以收定支、量入为出，开门办预算，加强政府债务风险防范。积极培育涵养税源、扩大税基，努力挖潜增收。继续深化投融资、招标采购管理体制改革。深化医药卫生体制改革，稳步推进公立医院改革试点，积极推进县级公立医院综合改革，建设区域卫生信息化平台。深化农村土地使用制度、集体经济组织产权制度改革，推动资源资产化、资产资本化。大力发展非公经济，实施初创企业、微小企业扶持工程和中小企业成长工程。

　　着力提升招商的有效性。进一步分清责任，明确界定县区、园区招商主体责任和市招商部门的组织、协调、指导责任，以及市直部门的服务责任。整合市政府驻外机构、招商分局、招商小组资源，划分区域负责招商与联络，将专业招商小组落到载体单位，实行项目个人领衔、小组负责。加强专业招商，全方位开展产业招商，积极开展股权招商、产权招商和平台招商。深入细致地做好招商前期工作，不断优化投资环境，实现招商引资良性循环。深化与长三角等地区的合作，重点加强与南京的深度合作，稳步推进马芜同城化，研究谋划与合肥合作，深化与宿州结对合作。

　　增强开发园区集聚带动力。坚持有所为、有所不为，进一步明确各园区功能定位，重点发展1-2个主导产业，加快形成分工科学、产业集聚、错位互补、共同发展的园区发展格局。市开发区重点发展汽车及零部件制造业和电子信息产业，与和县加快共建郑蒲港新区。博望新区重点发展以刃模具、剪折机床为主的机械装备制造业，力争省级高新区创建取得新成效。慈湖高新区重点发展机械装备制造、精细化工业，与和县加快共建乌江工业园，力争早日晋升为国家级高新区。示范园区重点发展现代装备制造业和新能源产业，积极打造战略性新兴产业集聚区。滨江新区重点发展现代服务业、文化产业。秀山新区重点发展软件及服务外包、金融、总部经济。统筹推进其他开发园区扩容升级。

　　(五)致力重点突破，推动现代服务业提速发展。按照建设“江海联运枢纽中心”、“运动健康休闲度假中心”的要求，加快发展以物流为重点的生产性服务业和商贸、文化、旅游、体育相结合的生活性服务业，服务业增加值增长11%以上。

　　积极谋划“一基地两园区”建设。立足港为城用，城以港兴，港城互动，做活“港”文章，对境内长江岸线资源统一规划、建设、管理，以郑蒲港为依托，以铁矿石、煤炭交易集散为重点，谋划打造港口物流基地。依托马钢等企业，以钢铁原材料及成品、半成品加工销售为重点，充分发展现货、期货交易，谋划打造钢铁交易产业园。利用华菱、星马及周边汽车产业集聚的有利条件，引进发展汽车核心零部件，谋划打造汽车及零部件交易产业园。

　　改造提升商贸流通业。加快推进金鹰天地广场、亿丰环球购物中心项目建设，不断增强城区主商圈辐射带动力。适应新的消费观念，着力引进新型营销模式，培育品牌直销购物中心。积极发展新型流通业态，继续实施“万村千乡市场工程”和“新网工程”。大力发展服务贸易和服务外包，启动综合保税区申报工作，提高口岸通关效率。稳定现有出口市场，积极开拓新兴市场；引导支持企业“走出去”，带动成套设备和材料出口。

　　促进旅游业提速发展。整合一江两岸旅游资源，完善全市旅游发展规划，做好含山、和县区域旅游规划，串点成线，推出成熟而具有特色的旅游产品。完成江心洲总体对外招商，推进濮塘休闲度假区、香泉昭关温泉旅游度假区、李白旅游景区、凌家滩文化旅游区等项目建设，叫响“温泉之乡”、“蔬菜之乡”、“诗歌之城”、“山水生态之城”旅游品牌，全年旅游总收入突破100亿元。

　　加快发展其他服务业。全年引进40家以上商务、中介服务机构，其中至少引进2家银行、2家基金管理公司，新设立10家小额贷款公司；至少引进规范的环评机构2家、会展服务机构3家、广告公司2家。稳定实质性贷款增长态势，确保新增贷款规模和增速保持全省前列。积极发展企业债券、集合票据、私募股权投资等融资工具。规范金融秩序，防范金融风险。围绕产品、产业和地方特色，大力发展会展经济，全年举办各类展会50场次以上，积极参加国内外重要展会。

　　(六)推进城乡统筹，促进县域经济加快发展。坚持工业化、城镇化、农业现代化“三化”同步，工业园区、现代农业产业园区、农村新社区“三区”联动，推动县区进一步增强综合实力，在全省争先进位，确保固定资产投资、财政收入平均增幅30%以上。支持县区培育做大主导产业。强化工业强县、特色就是核心竞争力的理念，充分发挥县区各自产业优势，形成特色鲜明、充满活力的县域经济发展格局。含山县重点发展精密铸造、制冷配件、建材陶瓷产业；和县重点发展泵阀制造、精细化工业；当涂县重点发展装备制造、新材料、生物医药、食品加工业。花山区重点发展现代服务业，力争成为省级服务业综合改革试点区、省级旅游度假区；雨山区重点发展装备制造、再生资源产业，努力打造国家级动漫游戏基地；金家庄区重点发展装备制造、电力能源、造纸印刷产业，着力建设沿江工业带。加快发展现代农业。立足于打造绿色食品供应中心，大力推进农业规模化、产业化、特色化、品牌化发展。力争农村土地流转面积占总承包经营面积30%以上，进一步提高规模种植、养殖比重。加快推进当涂现代农业示范区、博望现代农业示范园、和县台湾农民创业园、含山油脂和棉纺产业加工示范区等农业园区建设。大力推进农业产业化，做大做强蒙牛乳业、达利食品、雨润肉制品、黄池食品等龙头企业，支持大平粮油上市融资，新增年营销收入超亿元企业3家以上。发挥和县蔬菜产业基础优势和蔬菜博览会品牌效应，加快建成全国重要的蔬菜集散地和全省最大的蔬菜出口基地；整合当涂水产、含山粮油资源，打造全国知名绿色生态品牌。

　　改善农村生产生活条件。坚持交通先行，加快省道、县乡公路改造步伐。完善村镇建设规划，促进工业向园区集中、人口向城镇集中、土地向规模集中、生产要素向重点区域集中。推进省市新农村试点镇村建设，进一步增强示范效应。实施“水利安徽”战略，谋划推进一批重点水利工程建设项目。深入推进农村危房改造，扎实开展农村环境连片整治，积极实施垃圾集中处置工程。加大农村公共服务资源配置力度，推动优质公共服务资源向农村延伸。重点加强农民就业技能培训，造就更多适应工业化、城镇化和农业现代化需要的新型农民。

　　(七)创建国家公共文化服务体系示范区，推动文化大发展大繁荣。深入贯彻党的十七届六中全会精神，深化文化体制改革，大力发展文化事业和文化产业，努力建设文化强市。

　　加强社会主义核心价值体系建设。深入开展社会公德、职业道德、家庭美德、个人品德教育，在全社会形成积极向上的精神追求和健康文明的生活方式。大力弘扬“聚山纳川、一马当先”的新时期马鞍山精神，深入推动思想解放、观念更新。坚持抓好群众性精神文明创建工作，促进城乡文明一体化。

　　大力发展文化事业。按照公益性、基本性、均等性、便利性的要求，加强公共文化设施建设，满足人民群众基本文化需求，完成国家公共文化服务体系示范区创建任务。加快推进李白文化博览园建设，实施烟墩山、和县猿人遗址保护规划，积极申报当涂民歌成为国家非物质文化遗产示范项目，争取太白楼晋级国家文物保护单位。全面发展网络传媒、广播电视、新闻出版、文学艺术事业，大力实施精品战略，积极开展群众性文化活动。

　　加快发展文化产业。加快推进洪滨丝画产业园、艺谷产业园、千字文文化园、古床博物馆、明清建筑园和小九华寺改建，规划建设影视基地。引导社会资本以多种形式投资文化产业，培育一批骨干文化企业。积极发展新兴文化业态，申报争取国家级动漫企业2家。深入推进文化体制改革，健全文化产品和要素市场，提高文化资源市场化配置水平。

　　(八)坚持民生为先，进一步加强和创新社会建设与管理。积极而为、量力而行、循序渐进，加大民生领域投入，切实办好涉及民生的大事要事。实施40项民生工程，投入资金30亿元，增长22%。加强创业就业和社会保障工作。实施居民收入倍增规划，加快构建居民收入持续较快增长的多元支撑体系。实施更加积极的就业促进政策，确保城镇新增就业3.4万人。坚持以创业带动就业，扎实做好创建创业型城市迎检工作。全面建立城乡居民养老保险制度。抓好三县新型农村养老保险全国试点，全市参保率保持在80%以上。完善城乡居民最低生活保障和救济制度，大力发展社会福利、社会救助、社会优抚和慈善事业。全力推进保障房建设，继续加大安置房建设力度，努力实现由“人等房”向“房等人”的转变。全面完成老旧小区三年整治计划。实施“渔民上岸”工程。重视做好稳定物价工作。

　　加快发展各项社会事业。坚持优先发展教育，实施学前教育三年行动计划，提高初中办学水平，促进教育公平和均衡发展。中等职业教育园建成使用。努力建设更具特色的职业教育和更高质量的高等教育。推进基本公共卫生服务逐步均等化，建立市级综合性医院与基层医疗机构分工协作、分级诊疗、双向转诊机制，构建市县乡一体医疗服务体系。继续稳定低生育水平，有效遏制出生人口性别比偏高趋势，提高出生人口素质。推进全民公共体育服务体系建设，大力开展全民健身活动。续建市体育会展中心。确保“三馆”开馆运行。强化服务业统计，推行企业一套表制度。建设城乡气象防灾减灾保障工程。维护妇女、儿童和老年人合法权益。加强国防后备力量和人民防空建设。维护民族团结和宗教和谐稳定。充分发挥工会、共青团、妇联、科协、文联、社科联、侨联、残联等人民团体的作用。

　　提高社会管理水平。加强社区建设，完成社区建设发展规划编制，推行社区扁平化、网格化管理，引导鼓励社会资金参与社区服务。加强社会治安综合治理，加快推进社会治安防控体系建设，着力提高打击犯罪效能，统筹推进科技强警和基础工作，不断增强群众安全感。巩固信访综合治理成果，进一步畅通信访渠道，完善三级信访平台，实现案结事了。深入开展医患、校生纠纷综合治理和重点工程保平安活动。推进户籍管理制度改革，加强进城务工人员服务与管理。提升食品药品安全保障能力和水平。认真落实安全生产责任制，坚决杜绝重特大安全事故发生。进一步提高突发事件应急处置能力。

三、进一步加强政府自身建设

　　马鞍山已进入转型发展、加快发展的关键时期，新形势新任务对政府工作提出了新的更高要求，必须通过改革创新进一步加强自身建设。

　　(一)勤政为民，建设责任政府。把建设好马鞍山、让马鞍山人民安居乐业作为各级政府最大的责任，忠诚人民，忠诚这座城市。始终保持昂扬向上、奋力赶超、争创一流的精气神，坚定必胜信念，千方百计做大经济体量，提高发展质量。分清市场、政府和群众的主体责任，不越位、不缺位、不错位。强化县区、园区、新区经济发展主体责任，一级对一级负责；相信基层、依靠基层、服务基层，一级为一级服务。政府全体工作人员要敢于担责，敢于负责，一切从有利于工作出发，不做老好人，不怕得罪人，做到“我的岗位我负责，我的工作请放心”。

　　(二)与时俱进，建设开明政府。适应区划调整变化，从“偏江一隅”视角转到“一江两岸”拥江发展的视角，在更大空间、更大范围思考和谋划马鞍山发展。适应经济体制变化，把握好市场和计划的概念，树立市场经济是区域经济、竞争经济、智慧经济的理念，自觉按照市场的思维思考问题，按照市场的办法解决问题。适应宏观形势变化，关注宏观经济形势和政策走向，灵活应对，趋利避害。认真钻研业务，增强推动科学发展的能力和本领，做到工作说得清、问题讲得明、解决有对策。做工作要讲成本，既会算长账、算大账，也会算细账。带头讲诚信，想明白了再说，说了就要做，做的要比说的好。

　　(三)依法行政，建设法治政府。主动接受人大的法律监督、工作监督和政协的民主监督，完善政府工作规则，扩大政务公开，深入推进法治政府建设。注重加强制度建设，利用问题反推机制，查找不足，建章立制，努力让主观、人为的因素少一些，让客观、制度的因素多一些。政府全体工作人员要有强烈的依法办事意识，一切按法律办，按制度办，按规矩办，在法律法规和制度面前不能心存侥幸。认真研究法律法规，熟练运用政策和规则，有效推动工作。加强理想信念和廉洁从政教育，严格落实党风廉政建设责任制，全面推进公共权力规范运行预警机制建设。加强行政监察和财政、审计监督，做到绩效有评估、过失必追究。

　　(四)改进作风，建设务实政府。把强化落实作为重中之重，把落实体现在狠抓每一天、每件事、每个项目上。按照“完成任务，不出问题，群众满意，领导认可”的要求，任务一件一件地分解，项目一项一项地抓，问题一个一个地解决。投入力量时间，推动工作快速落实，做到今日事今日毕，不留欠账，不打折扣。规范政府工作流程，推行扁平化管理，完善重点工作领衔负责制，下基层到一线，点对点开展工作。健全目标管理考核，加强对落实过程的督促检查，看进度、查问题、促整改。分级负责、花钱预算，勤俭节约、精打细算，把有限的资金和资源用在发展经济、改善民生上，以实实在在的业绩取信于民。

　　各位代表！建设新马鞍山、大马鞍山的大幕已经拉开，形势催人奋进，前景令人期待！让我们在市委的坚强领导下，紧紧依靠全市人民，齐心协力，开拓创新，大干快上，圆满完成本届政府各项目标任务，为加快转变经济发展方式、推动一江两岸协调发展、建设更高水平小康社会奠定坚实基础，以优异成绩迎接党的十八大胜利召开！
